# High Throughput Sequencing of *Entamoeba* 27nt Small RNA Population Reveals Role in Permanent Gene Silencing But No Effect on Regulating Gene Expression Changes during Stage Conversion, Oxidative, or Heat Shock Stress

**DOI:** 10.1371/journal.pone.0134481

**Published:** 2015-08-06

**Authors:** Hanbang Zhang, Gretchen M. Ehrenkaufer, Dipak Manna, Neil Hall, Upinder Singh

**Affiliations:** 1 Division of Infectious Diseases, Department of Internal Medicine, Stanford University School of Medicine, Stanford, California, United States of America; 2 Center for Genomic Research, Institute of Integrative Biology, Biosciences Building, University of Liverpool, Liverpool, United Kingdom; 3 Department of Microbiology and Immunology, Stanford University School of Medicine, Stanford, California, United States of America; Centro de Investigacion y de Estudios Avanzados del Instituto Politecnico Nacional, MEXICO

## Abstract

The human parasite *Entamoeba histolytica* has an active RNA interference (RNAi) pathway with an extensive repertoire of 27nt small RNAs that silence genes. However the role of this pathway in regulating amebic biology remains unknown. In this study, we address whether silencing via 27nt small RNAs may be a mechanism for controlling gene expression changes during conversion between the trophozoite and cyst stages of the parasite. We sequenced small RNA libraries generated from trophozoites, early cysts, mature cysts, and excysting cells and mapped them to the *E*. *invadens* genome. Our results show that, as in *E*. *histolytica*, small RNAs in *E*. *invadens* are largely ~27nt in length, have an unusual 5'-polyphosphate structure and mediate gene silencing. However, when comparing the libraries from each developmental time-point we found few changes in the composition of the small RNA populations. Furthermore, genes targeted by small RNAs were permanently silenced with no changes in transcript abundance during development. Thus, the *E*. *invadens* 27nt small RNA population does not mediate gene expression changes during development. In order to assess the generalizability of our observations, we examined whether small RNAs may be regulating gene expression changes during stress response in *E*. *histolytica*. Comparison of the 27nt small RNA populations from *E*. *histolytica* trophozoites from basal conditions, or after heat shock or exposure to oxidative stress showed few differences. Similar to data in *E*. *invadens* development, genes targeted by small RNAs were consistently silenced and did not change expression under tested stress conditions. Thus, the biological roles of the 27nt small RNA population in *Entamoeba* remain elusive. However, as the first characterization of the RNAi pathway in *E*. *invadens* these data serve as a useful resource for the study of *Entamoeba* development and open the door to the development of RNAi-based gene silencing tools in *E*. *invadens*.

## Introduction

The anaerobic protozoan parasite *Entamoeba histolytica* is the causative agent of amebiasis, a major health problem in developing countries and a leading cause of mortality due to parasitic infection [1]. The parasite has two life stages: the cyst form, which does not undergo division but can survive in the environment and is responsible for disease transmission, and the trophozoite, a motile, proliferative form which can cause colitis as well as extra-intestinal infections in humans [2]. Infection begins when cysts are ingested; excystation to trophozoites occurs in the small intestine, and subsequently colonization and/or infection by trophozoites is established in the colon [2]. Due to unknown triggers, trophozoites in the colon convert to cysts, which are shed in the stool and propagate the infectious cycle by infecting a new host [3]. Thus, inter-conversion between the cyst and trophozoite developmental forms is required for intestinal colonization and tissue invasion, as well as disease propagation.

Due to the absence of an *in vitro* model for induction of encystation in *E*. *histolytica*, most research on *Entamoeba* development has been performed using the related reptilian parasite *E*. *invadens*, in which both encystation and excystation can be performed with high efficiency *in vitro* [4, 5]. Using this system, many aspects of *Entamoeba* development have been described, including the recent publication of two studies identifying changes in the transcriptome throughout development. Significant changes in transcript abundance were noted with ~50% of genes changing expression in at least one time-point during encystation and excystation [6, 7]. Importantly, transcriptional regulation is a conserved theme in *Entamoeba*, as heat shock and oxidative stress responses are also associated with significant transcriptional changes [8, 9]. These findings provide the motivation to better understand the mechanisms regulating gene expression changes during development and stress in *Entamoeba*.

RNA interference (RNAi) is a common gene regulatory mechanism in many eukaryotes, relying on short (~22–30nt) RNA molecules to effect either transcriptional or post-transcriptional gene silencing [10]. The RNAi pathway controls multiple aspects of biology including developmental changes, anti-viral defense response, and maintenance of genome integrity via transposon silencing [10–12]. Some RNAi pathway genes are conserved in *E*. *histolytica* and ~27nt small RNAs with an unusual 5'-polyP structure have been cloned from *E*. *histolytica* trophozoites [13, 14]. These small RNAs largely map antisense to genes and mediate transcriptional gene silencing via a nuclear localized Argonaute protein [13]. Furthermore, we have shown that small RNAs regulate some strain-specific gene expression patterns in *E*. *histolytica* [13, 14]. RNAi pathway proteins such as Argonaute and RNA-dependent RNA polymerase are conserved in other *Entamoeba* species, including *E*. *invadens* and *E*. *dispar*, further supporting the idea that this mechanism is important for *Entamoeba* biology [7, 14]. However, the roles of small RNAs in regulating gene expression changes under different biological conditions are not currently known.

In this paper, we seek to determine whether 27nt small RNAs mediate regulation of gene expression during *Entamoeba* development or stress response. In model systems, micro RNAs (miRNAs) and small interfering RNAs (siRNAs) have been implicated in diverse biological processes including cardiac development [15], regulation of flowering in *Arabidopsis* [16], oxygen response during angiogenesis [17] and phosphate deficiency [18]. Additionally, in plants siRNAs are involved in responses to heat and salt stresses [19]. To see if *Entamoeba* small RNAs may play some similar roles, we generated and sequenced 27nt small RNA libraries from four stages of encysting and excysting *E*. *invadens* parasites, as well as from *E*. *histolytica* trophozoites subjected to oxidative stress or heat shock. In this first survey of small RNAs in *E*. *invadens* we find that *E*. *invadens* has an extensive population of 27nt small RNAs with a 5'-polyP structure, which are derived from many genomic locations including silenced gene loci—findings that recapitulate observations in *E*. *histolytica*. However, analysis of 27nt small RNA profiles under encystation and excystation conditions (as well as *E*. *histolytica* small RNA libraries from heat shock or oxidative stress) indicate that overall there are very few changes in the 27nt small RNA populations under these conditions. Consistent with this observation, we found few changes in transcript abundance for genes targeted by small RNAs, during either development or stress responses. Therefore, we conclude that it is unlikely that the 27nt small RNAs are playing a direct role in regulating the transcriptional changes underlying development, oxidative stress or heat shock response. This is an unexpected result given the robust maintenance of this pathway in *Entamoeba* and indicates that further studies will be needed to determine the endogenous biological roles for the RNAi pathway in *Entamoeba*.

## Methods

### Parasite culture and induction of development and stress conditions

*E*. *invadens* strain IP-1 was cultured in LYI-S-2 at 25°C as previously described [7]. For encystation, we followed previously published methods [7]. Trophozoite cultures were chilled, pooled, washed, and then split into six T25 flasks in encystation media (47% LYI-LG + 7% adult bovine serum). For each time point (24h or 72h) two flasks of encysting parasites were collected. Mature cysts (72h) were additionally treated with 0.1% sarkosyl prior to RNA preparation to lyse remaining trophozoites. For excystation, we used the protocol as in [7]. Two flasks of mature 72h cysts were incubated overnight in distilled water at 4°C, which lyses any remaining trophozoites and then placed in excystation media (LYI-LG with the addition of 1mg/ml bile, 40mM sodium bicarbonate, 1% glucose and 10% serum) for 8h. Encystation efficiency was monitored by calculating the percent survival after treatment by 0.1% sarkosyl for 30 minutes at 4°C. For library construction, we used one experiment for all four time-point samples, with an overall encystation efficiency of 68%.

For stress conditions, we used previously published methods with H_2_O_2_ stress (1mM H_2_O_2_ for 1hr) [8] and heat-shock (42°C for 1hr) [9]. Starting from one T25 culture flask, 18 tubes (16ml) of *E*. *histolytica* HM-1:IMSS cultures were set up, and grown for 2 days at 37°C. On the day of treatment, the tubes were divided into three groups of 6 tubes each. One group was used as an untreated control, one group for H_2_O_2_ exposure, one group for 42°C heat shock. After treatment, all tubes were iced and total RNA for each condition prepared using Trizol (Life Technologies). We performed three biological replicates, pooled samples from all biological replicates, and made one library using pooled RNA for each condition.

### Total RNA prep and Capping assay

Total RNA for all *E*. *invadens* development samples was prepared using a modified Trizol protocol, which involved using French press to break mature cysts [7]. For the capping assay, the ScriptCap m7G capping system (Epicentre) was used [14]. After treatment, samples were phenol:chloroform extracted, ethanol precipitated, labeled with [5’-^32^P]-pCp and resolved on a 12% polyacrylamide gel. Gels were exposed to a phosphor screen and imaged on a Personal Molecular Imager (Bio-Rad).

### Western blot, Immunofluorescence assay (IFA) and Cell fractionation

For Western blot and IFA analyses, the same custom-made polyclonal antibody to EHAGO2-2 from our previous report [20] was used. For Western blot analysis, primary antibody was used at 1:2500 dilution in 2.5% milk of PBST solution for overnight at 4°C. The secondary anti-Rabbit HRP (Jackson labs) was used as a 1:10, 000 dilution in 2.5% milk PBST solution for 1 hr at room temperature. Anti-Actin antibody is from MP Biomedicals, and anti-Histone H3 is from Abcam. For IFA, we used the same protocol as in [20] with the following changes: after fixation with Acetone: Methanol (1:1), cells were permeabilized with 0.1% TritonX-100 for 10 min. Primary antibody was used at a 1:250 dilution in 1% BSA-PBS and incubated at 4°C for overnight followed by Alexa Fluor 488-conjugated anti-rabbit secondary antibody (1:2500; Molecular Probes) for 1 hr at room temperature. *E*. *invadens* parasites were prepared for cytosolic fraction and nuclear fraction using our previously published protocol [21].

### Reverse Transcriptase PCR (RT-PCR)

For cDNA preparation: 2μg total RNA from each time point was treated with DNase I as in [22]. The RNA was then separated into two aliquots (minus-RT and plus-RT), and cDNA were made using SuperScript III First-Strand Synthesis System kit (Invitrogen). 2μl of cDNA was then used as a template for PCRs and all PCR reactions were 30 cycles. Primers are: EiAGO2-2 forward ATGGCGCATATCCAAAGAAG, reverse CGCTGATTGGTTGATGTTTG; EIN_192230 forward AACAAGTCGGGTTGGTCTTG, reverse CTCGAATGTTCCAGCAGTCA; EIN_040930 forward TGATTGAAGCTGGGTGGGAT, reverse GCAATGGAAGAACAACGGGT; EIN_099680 forward CAGGTTCTGAGCGACAACAA, reverse CGCATTTTTCCACCTCTGAT; EIN_186850 forward GCCACGAAAAATCCAAAGAA, reverse GTTGCACTCCTCGCTCTTTT; EIN_202650 forward AAAGCCCATGCAAAATCATC, reverse TTGCAATTTGCTTTCCACAC.

### Library construction and sequencing

We used the NEBNext multiplex small RNA library prep set for Illumina (NEB#E7300S) and followed the manufacturer’s protocol with the following modifications: total RNA (25–50 μg) was fractioned to 15–30nt size using 12% polyacrylamide urea gel. The size-selected RNA was treated with tobacco acid pyrophosphatase [TAP] to convert 5’ poly-P to 5’ single-P (Epicentre). After cDNA generation, barcode primers were PCR incorporated into each sample. We typically used 8–12 PCR cycles to get a final PCR product. The final PCR product was quantified using Nanodrop and samples were pooled for Illumina sequencing using the MiSeq platform. The number of reads generated for each library is listed in Tables 1 and 2.

**Table 1:** Composition of small RNA libraries from *E*. *invadens*. **(A)** Sequenced libraries from each time-point during development were processed to remove barcodes, size selected for reads >15nt or <40nt, duplicate reads removed and the remaining unique reads sequentially mapped against each sequence type shown. Note that a small number of reads mapped both antisense and sense to transcripts, hence the total number of reads mapped to “transcript” is lower than the total number of reads mapping either antisense or sense. Total number of reads and percent of unique mapped reads are shown.

Library	Trophozoite	% of unique reads	Early Cyst (24h)	% of unique reads	Mature Cyst (72h)	% of unique reads	Excystation (8h)	% of unique reads
**Total reads**	**458,456**		**1,883,073**		**1,256,373**		**1,883,410**	
**Unique reads**	284,793		817,851		682,981		593,686	
**Map to tRNA**	2,465	0.9%	4,879	0.6%	4,944	0.7%	5,132	0.9%
**Map to rDNA**	16,908	6%	11,743	1%	10,397	2%	18,286	3%
**Map to TEs and REs**	45,376	16%	120,377	15%	102,500	15%	89,096	15%
**Map to genome**	**135,654**	**48%**	**353,052**	**43%**	**307,565**	**45%**	**269,447**	**45%**
**Map to transcripts**	**47,218**	**17%**	**130,753**	**16%**	**114,043**	**17%**	**100,455**	**17%**
**Map antisense to genes**	31,971	11%	84,156	10%	75,209	11%	63,554	11%
**Map sense to genes**	15,274	5%	46,695	6%	38,942	6%	36,959	6%
**Map to intergenic regions**	88,436	31%	222,299	27%	193,522	28%	168,992	29%

**Table 2:** Mapping of *E*. *invadens* small RNAs to transposons and repetitive elements. Unique reads from *E*. *invadens* trophozoites, after removal of tRNA and rRNA mapped reads, were aligned to each type of transposable and repetitive element found in the *E*. *invadens* genome. Total read number, percent of unique reads, and percent of the *E*. *invadens* genome that each element represents (adapted from [30]) are shown.

Element type	Unique read number (Trophozoite)	% of unique reads	% of genome
**Ei_DDE**	22,965	8.1%	4.1%
**Ei_ERE**	835	0.3%	0.4%
**Ei_hAT**	3,156	1.1%	1.1%
**Ei_LINE**	1,619	0.6%	0.1%
**Ei_mariner**	9,624	3.4%	1.0%
**Ei_MuDR**	5,667	2.0%	1.3%
**Ei_piggyBac**	563	0.2%	0.1%
**Ei_Polinton**	947	0.3%	0.8%

### Mapping of reads

Raw Illumina sequences were pre-processed to remove barcodes, converted to FASTA format and size selected to remove all sequences <15 and >40nt. Duplicate reads were removed and all downstream analysis was performed using unique sequences. Mapping to the *E*. *invadens* or *E*. *histolytica* genome, repetitive elements, ribosomal DNA, tRNA and transcripts was performed using Bowtie v0.12.5 [23] (bowtie-bio.sourceforge.net) with the parameters:-v 0—all. A schematic of the alignment methodology is shown in S1 Fig Genome, transcript, and protein sequences were downloaded from amoebadb.org; version 1.3 for both genomes. Repeat sequences were extracted from the *E*. *histolytica* or *E*. *invadens* genome with Bedtools [24] (FastaFromBed), using coordinates provided by Lis Caler and Hernan Lorenzi (personal communication). For trophozoites, a total of 458,456 reads were sequenced, of which 284,793 (62%) were unique; this high percentage of unique reads indicates that the sequencing is not saturated, and further sequencing would be required to identify rare species. Unique small RNA reads were then aligned to tRNA and ribosomal RNA; these reads were removed from further downstream analysis.

### Analysis of small RNA composition

For each small RNA library, the length profile and nucleotide distribution at each position were determined in R using the ShortRead package [25]. To determine the pattern of small RNA distribution on gene loci, each small RNA sequence was assigned a position value based on the position of the starting nucleotide along the gene, normalized by gene length and histograms showing the frequency at each position were plotted. A similar strategy was used for plotting small RNA mapping to promoters. Small RNA alignment of each gene with ≥20 antisense RNAs was performed against the region extending from -100 to +100 (relative to ATG). Libraries were tested for presence of miRNAs using the program mirDeep2 v. 0.0.7 [26] with a reference miRNA database. Sequence homology was determined using BLASTP (downloaded from http://blast.ncbi.nlm.nih.gov) with an e-value cutoff of < 1e^-20^.

## Results

### Generation of a 27nt small RNA library from *E*. *invadens* trophozoites

**Fig 1:**
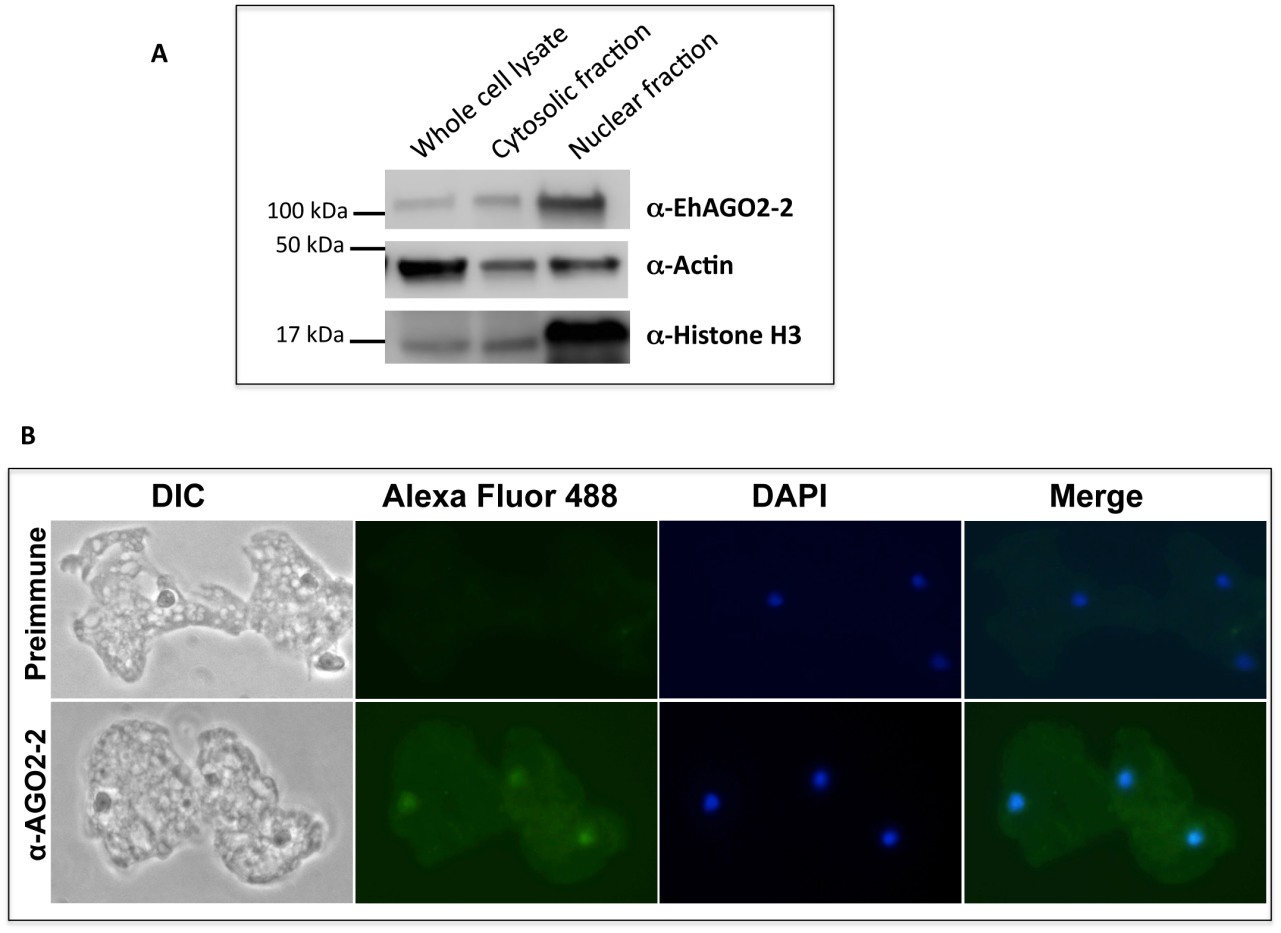
A custom-made antibody to *Entamoeba* AGO2-2 detects a 110kDa protein, which is enriched in the nucleus. **(A)** Western Blot detects a band at 110 kDa using α-EhAGO2-2 antibody on *E*. *invadens* whole cell lysate, cytosolic fraction and nuclear fractions. **(B)** Immunofluorescence assay on *E*. *invadens* trophozoites showed nuclear enriched signal, which co-localizes with DAPI. Some cytoplasmic signal is also noted.

The *E*. *invadens* genome is significantly larger than that of *E*. *histolytica* (~40MB versus ~20MB); however, they are both AT rich and have large gene families [7, 27]. The current *E*. *invadens* genome contains multiple RNAi pathway genes: four genes encoding full or partial Argonaute proteins, two genes encoding RNA-dependent RNA polymerase (RdRP), and an RNaseIII domain containing protein [7]. Several of these genes have developmentally regulated expression, including an RdRP gene (EIN_181590) that has increased expression early in encystation and an Argonaute gene (EIN_033570) with very high expression during excystation [7]. These data suggest that the RNAi pathway is important in *E*. *invadens* biology and could play a role in regulating gene expression during stage conversion. To confirm the presence of Argonaute protein in *E*. *invadens*, we tested a custom-made anti-*E*. *histolytica* AGO2-2 antibody on *E*. *invadens* trophozoite lysate. In *E*. *histolytica*, the AGO2-2 protein is 110 kDa and is localized to the nucleus [20]. In *E*. *invadens* trophozoite lysate we identified a clear band at 110 kDa that is enriched in the nuclear fraction, indicating that a homologue of Argonaute 2–2 is present in *E*. *invadens* (Fig 1A). Bioinformatic analysis indicates that EiAGO2-2 is highly conserved (57% identical and 78% similar, compared to EhAGO2-2). Immunofluorescence assay revealed signal in trophozoites, which was enriched in the nucleus (Fig 1B). These findings are similar to the data for this protein in *E*. *histolytica*; thus, we conclude that the *E*. *invadens* AGO2-2 has nuclear localization and may have similar functions. To determine if levels of EiAGO2-2 transcript change during stage conversion, we performed RT-PCR on RNA isolated from trophozoites, early cysts (24h encystation), mature cysts (72h encystation) and excystation (8h after induction of excystation). We found that EiAGO2-2 transcript is expressed throughout development, although the expression level was reduced during encystation, relative to a control gene (S2 Fig). Whether this reduction has any functional consequences, or is simply due to reduced levels of AGO being required to maintain silencing in non-proliferative cysts is unknown at this point.

**Fig 2:**
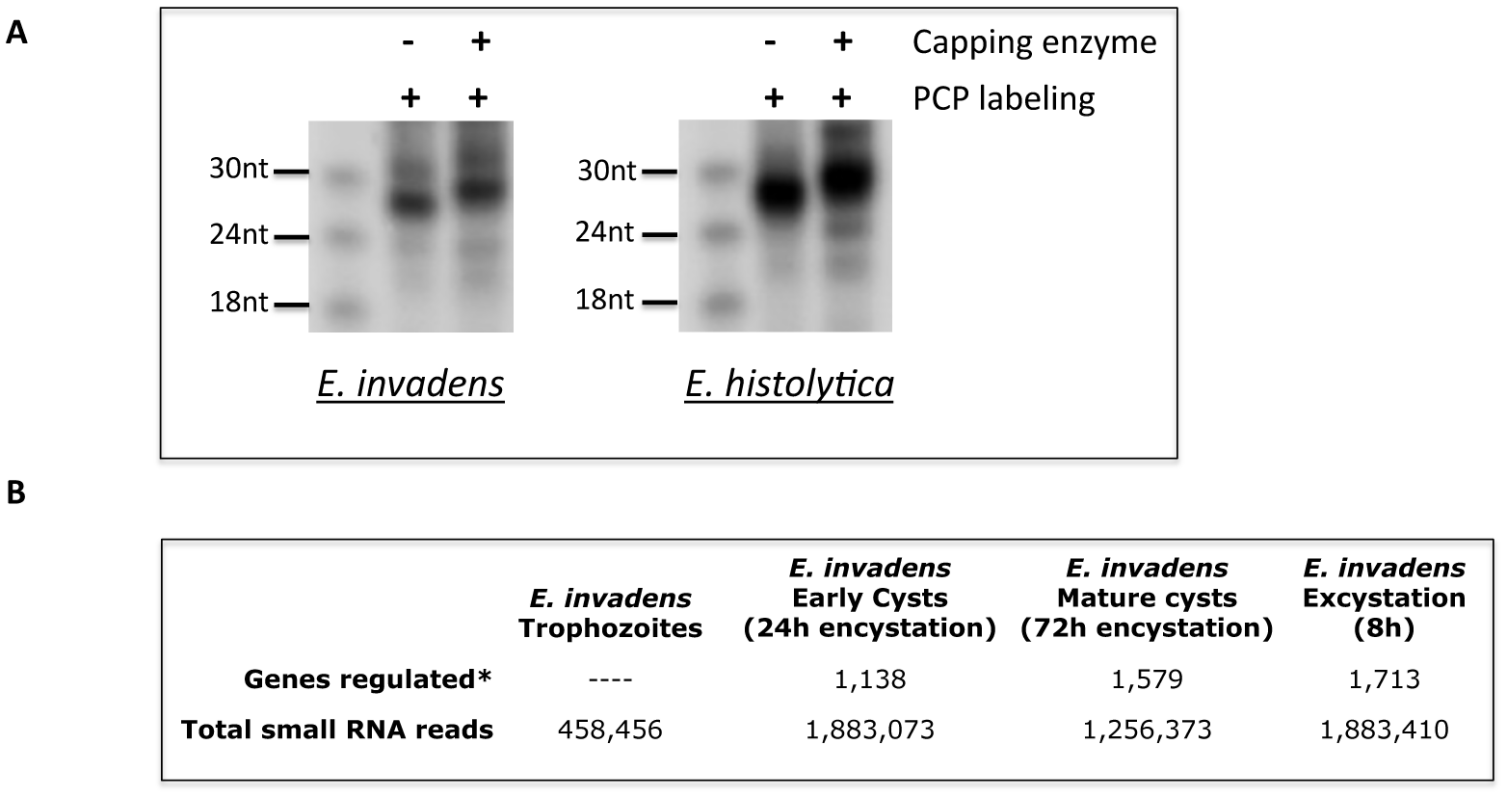
*E*. *invadens* small RNA biochemical analyses and small RNA library reads generated for each time point. **(A)** Biochemical analysis indicates that *E*. *invadens* small RNAs have 5'-polyphosphate and 3'-OH termini. Bulk preparations of *E*. *invadens* small RNAs are radioactively end-labeled with pCp, which indicates a 3'-OH terminus. Labeled RNAs are then treated with capping enzyme (which adds a guanosine cap to RNAs with a 5' polyP termini). Increase in the small RNA size from a bulk ~27nt population to a larger ~28nt population indicates that the *E*. *invadens* small RNAs have a 5'-polyphosphate termini. *E*. *histolytica* small RNAs are included as a positive control for characterization of 5’-polyP termini, as previously published [14]. **(B)** Extent of gene regulation and total small RNA reads at each time point of *E*. *invadens* stage conversion. The number of regulated genes during development are obtained from [7] and defined as genes with an FDR < 0.01. The small RNA reads are the total reads prior to size selection and removal of duplicates.

Previously we have noted that small RNAs in *E*. *histolytica* are 27nt in length, have 5' polyP termini and 3'-OH, and show a strong bias towards a G in the first 5'-nucleotide position [13, 14]. To determine if these features are conserved in *E*. *invadens*, we performed biochemical analysis on bulk small RNA populations from *E*. *invadens* trophozoites. Our results indicate that *E*. *invadens* small RNAs of ~27nt can be labeled with pCp and shift size upon treatment with capping enzyme (Fig 2A). Overall, these data indicate that the *E*. *invadens* 27nt small RNA population has a 5'-polyP structure and at least one free 3'-OH, similar to the structural features noted in *E*. *histolytica*. The 5'-polyP small RNAs cannot be primary products of Dicer processing but instead are similar to small RNAs in *C*. *elegans* and parasitic nematodes, which are derived from RdRP [28, 29].

In contrast to *E*. *histolytica*, in which the majority of mapped small RNAs were found to map to open reading frames (ORFs) [13], the largest category of small RNAs from *E*. *invadens* trophozoites mapped to intergenic regions (88,436 or ~31% of unique reads; compared to ~20% in *E*. *histolytica*) (Table 1). In addition, there was substantial mapping of small RNAs to retrotransposons and repetitive elements (45,376 or ~16% of unique reads; compared to ~4% in *E*. *histolytica*). There were significant numbers of reads that mapped to ORFs (~17%, compared to ~35% in *E*. *histolytica*) with the majority found in the antisense orientation (~68% of transcript derived reads). The existence of small RNAs mapping antisense to genes is similar to data from *E*. *histolytica* and given the analogy with that system, indicates that in *E*. *invadens* these small RNAs may also be involved in gene silencing. However, the substantial mapping of small RNAs to retrotransposons and repetitive elements suggests that in *E*. *invadens* the 27nt population may also have roles in maintaining genome integrity and stability, a function of the RNAi pathway noted in other systems but in contrast to our observations in *E*. *histolytica* [11–13].

**Fig 3:**
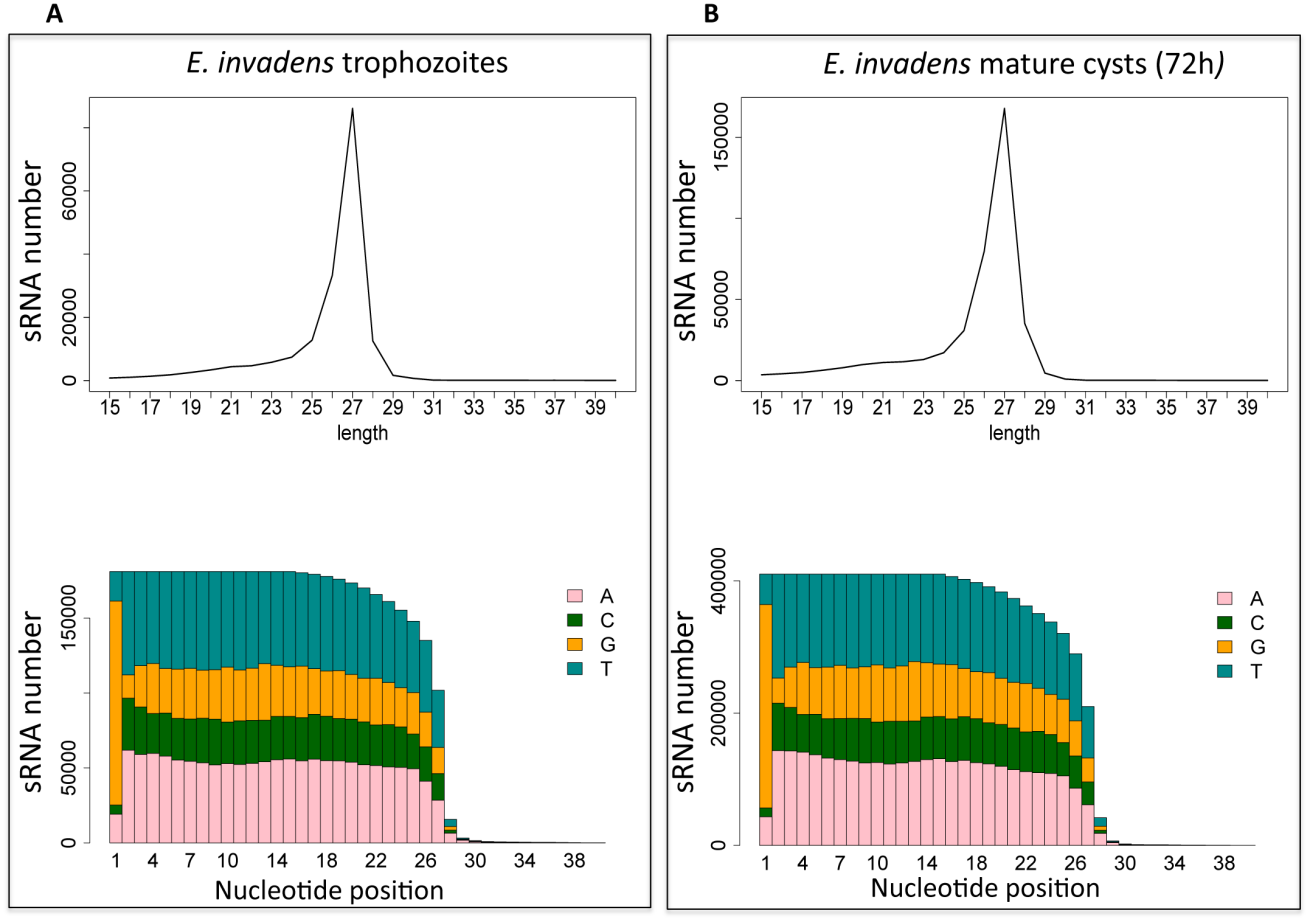
*E*. *invadens* small RNA length and nucleotide distribution. After removal of duplicates and size selection to remove RNAs >15nt or <40nt, a profile of length distribution and percent of each nucleotide base at each position were calculated for each library. Note the strong peak at 27nt in length and the preference for G at the 5' end in both datasets. (**A)** Trophozoite library, and (**B)** Mature cyst (72h) library.

**Fig 4:**
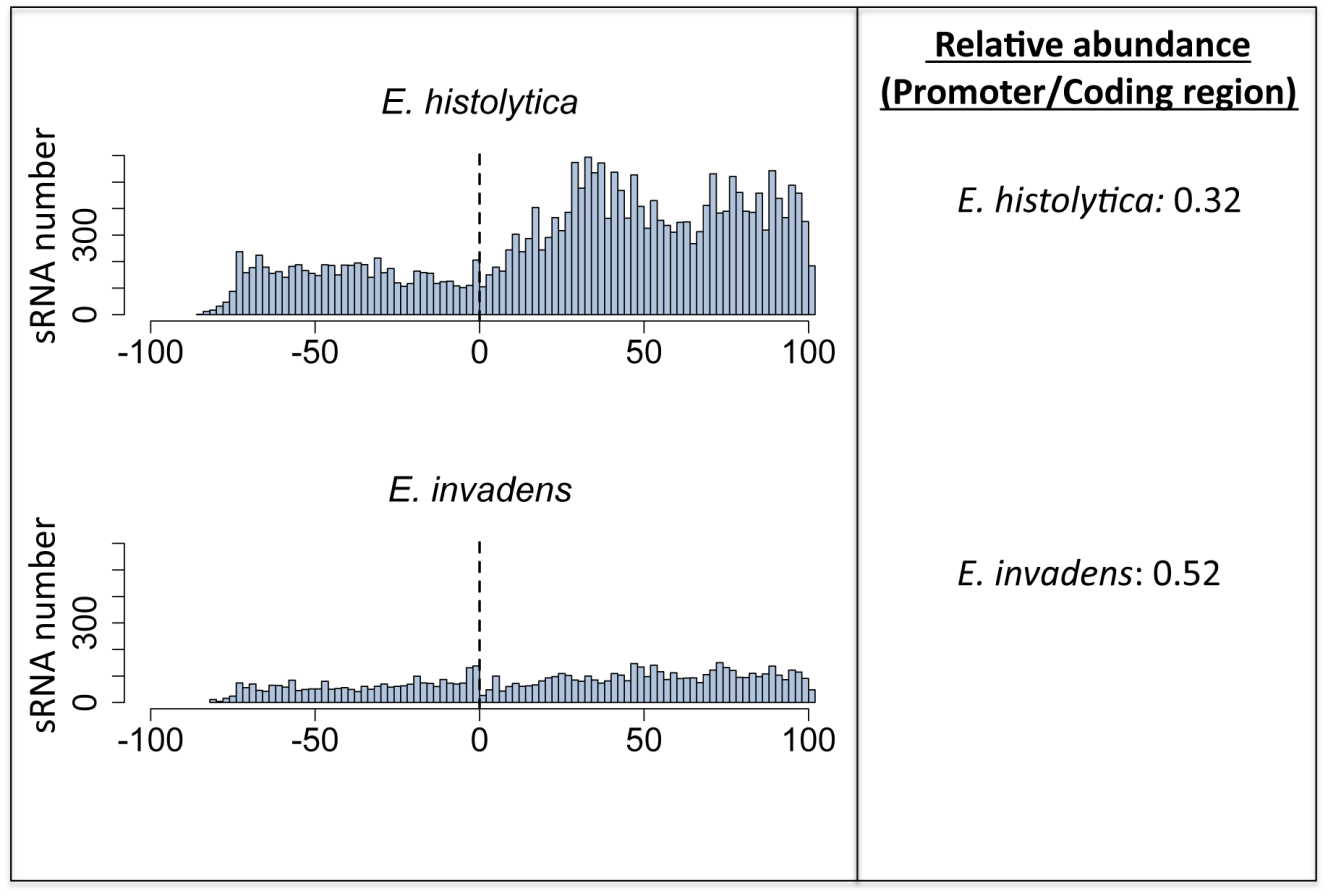
Mapped small RNAs in *E*. *invadens* show relatively greater occupancy of promoter regions compared to *E*. *histolytica*. Small RNA sequences from *E*. *histolytica* and *E*. *invadens* trophozoites were aligned to the region from -100 to +100 (relative to ATG) of each annotated ORF. The frequency of antisense RNA 5' ends (for genes with ≥20 antisense RNAs) at each position was plotted. The ratio of small RNAs in the upstream region (-100 to ATG) to small RNAs in the coding region (ATG to +100) of each ORF is shown.

Next, we analyzed the length and nucleotide composition of the small RNA reads from *E*. *invadens* trophozoites. There was a dominant peak in frequency of mapped reads at 27nt (Fig 3A), as well as a predisposition for 5'-G (Fig 3A), confirming the similarity between the *E*. *histolytica* and *E*. *invadens* 27nt small RNA population and suggesting that mechanisms for generation of small RNAs are conserved in these two species. However, there were some differences between the two populations. In *E*. *histolytica* the vast majority of antisense reads map to the 5' end of ORFs, while sense reads cluster at the 3'-end of ORFs [13]. This bias was only weakly observed in *E*. *invadens*, in which both sense and antisense reads showed a much wider distribution over the length of the mRNA (S3 Fig). We also noted that *E*. *invadens* small RNAs had greater mapping to promoter regions, in contrast to data in *E*. *histolytica* where small RNA abundance on coding regions is more pronounced (Fig 4). Whether the increased promoter occupancy by *E*. *invadens* small RNAs has regulatory roles is not known, although transcriptional gene silencing caused by siRNAs targeting promoter regions have been documented in yeast, fly and mammalian cells [31–33]. We additionally surveyed the small RNA library for evidence of potential miRNA species using mirDeep2 program, but found no definitive evidence of miRNA species.

### A subset of small RNAs map to repeat elements

Both *E*. *histolytica* and *E*. *invadens* have large numbers of transposable elements (TE) and repetitive elements (RE) in their genomes (~20% and ~10% respectively) [30]. However, the types of elements found in each species are distinct, with the *E*. *histolytica* genome largely populated by long and short interspersed nuclear elements (LINEs and SINEs), as well as the *Entamoeba* repetitive element (ERE) [30]. A more diverse collection of DNA transposons, including mariner and hobo-Ac-Tam3 (hAT) family elements, are found in *E*. *invadens* in addition to the LINEs, SINEs and EREs [30]. Given that a substantial percentage of small RNAs in *E*. *invadens* seem to derive from these elements, we decided to further examine their origins. We extracted sequences for each transposable and repetitive element in the *E*. *invadens* genome, and mapped the small RNA sequences against them. Small RNAs were found to derive from every type of TE/RE, including the ERE class, which was not found to be a source for small RNAs in *E*. *histolytica* (Table 2). These small RNAs are largely 27nt and map both sense and antisense to transposon transcripts. We tested for 5'-G bias and found that for all transposons >75% of small RNAs started with G (two representative examples are shown in S4 Fig), indicating that they are true small RNAs and not degradation products. The percent of small RNAs mapping to each class of transposable/repetitive element was generally proportional to the prevalence of the transposable element in the genome. The DDEs, which make up the single largest class of TEs, also had the highest number of small RNAs with ~50% of all transposon mapped small RNAs. However, there were some exceptions with LINEs (0.6% of all small RNAs but only 0.1% of the genome) being overrepresented and Polinton elements (0.4% of small RNAs and 0.8% of the genome) being underrepresented. The biological significance of this over/underrepresentation is unclear at present. In other parasites, such as *Trypanosoma brucei*, the majority of small RNAs derive from retrotransposons and transposon control is thought to be the main biological function of the RNAi pathway [34]. Although the abundance of transposon mapped small RNAs in *E*. *invadens* is intriguing, it remains to be seen whether they may play a similar biological role.

### Analysis of small RNAs during *E*. *invadens* development

In order to determine if the 27nt small RNAs control gene expression changes during *E*. *invadens* stage conversion, we generated small RNA libraries from *E*. *invadens* parasites at multiple time-points during development: early cysts (24h encystation), mature cysts (72h encystation) and excystation (8h after induction of excystation) and compared them to the trophozoite small RNA dataset obtained above. These time-points were chosen as we have previously generated transcriptome datasets from identical time-points [7], which allows the correlation of small RNA abundance with gene expression changes. Note that the early cyst (24h) and mature cyst (72h) samples were prepared from the same starting population of encysting parasites; hence, they represent points on a continuum. For mature cysts, samples were treated with 0.1% sarkosyl prior to RNA preparation in order to lyse remaining trophozoites, allowing us to harvest pure cyst material for library preparation and also to calculate the efficiency of encystation; 24h cysts were untreated as they are not fully sarkosyl resistant. Encystation efficiency was calculated by determining the percent parasite survival after sarkosyl treatment (at 72h after encystation) and found to be 68% for the encystation used for library generation. We were able to generate adequate small RNA libraries from all time points with a minimum of 400,000 small RNA reads in each library. The total number of reads sequenced for each time-point are shown in Fig 2B. The small RNA datasets from the encysting, mature cyst, and excystation time-points were processed using the same algorithm as the trophozoite sample. Neither the length distribution of the small RNAs or the 5'-G bias were affected by stage conversion (Fig 3B and S5 Fig), and as in the trophozoite sample, no potential miRNAs were found by mirDeep2 program. Alignment of the small RNA libraries from encysting and excysting parasites revealed that there were few significant differences between the relative proportion of small RNA reads mapping to each genomic region at any developmental time-point; the largest category in all time-points were small RNAs derived from intergenic regions (Table 1). Additionally, no significant changes in small RNAs derived from TEs occurred during development (S1 Table). The one type of small RNA that was significantly altered in abundance in development was that derived from ribosomal RNA, which oscillated from 5.9% of unique reads in trophozoites to low abundance in cysts (1.4% and 1.5% in 24h and 72h cysts, respectively), before rising again to 3.1% of unique reads during excystation. However, as these small RNAs are largely in the sense orientation, they are likely to be degradation products. Transcription of rRNA has been previously shown to fluctuate during encystation [35], slowing during early encystation but rising to trophozoite levels in mature cysts; the reduction in ribosomal derived small RNAs likely reflects these changes.

### Genes targeted by *E*. *invadens* small RNAs are silenced and do not change expression during development

In *E*. *histolytica*, antisense small RNAs play an important role in controlling gene expression and genes targeted by endogenous small RNAs have very low expression [13, 14]. Furthermore, triggering the production of small RNAs to a given gene can induce gene silencing [20, 21, 36]. We theorized that small RNAs may control gene expression during stage conversion. To determine if this was the case, we mapped small RNAs from each *E*. *invadens* library to coding sequences in the genome and identified all open reading frames (ORFs) to which ≥20 antisense (AS) small RNAs mapped. This cutoff (≥20 AS small RNAs) was chosen as the vast majority of genes with at least 20 AS small RNAs were not expressed, similar to data from *E*. *histolytica* [13].

**Fig 5:**
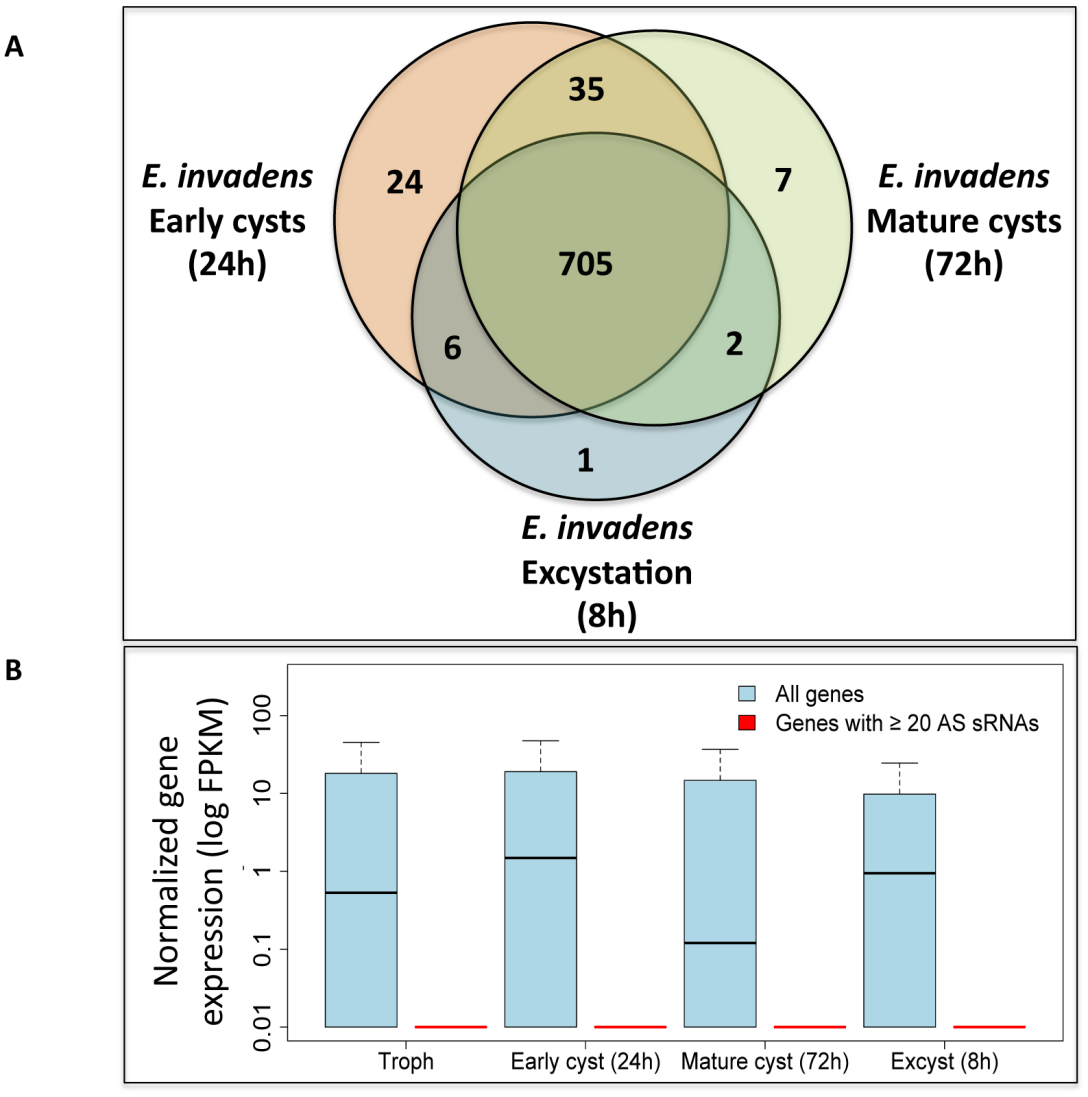
Comparison of genes with abundant small RNAs during *E*. *invadens* development. **(A)** Venn diagram of genes with ≥20 AS small RNAs mapped at each time-point. Note that the majority of genes with small RNAs are represented in all time points. Due to the smaller number of reads in the trophozoite library, all genes with ≥20 small RNAs in that library are present in the other libraries; hence this time point was not included in the diagram. **(B)** Genes with ≥20 AS small RNAs are not expressed during development. Expression data are based on RNA-Seq from *E*. *invadens* trophozoites, early cysts (24h), mature cysts (72h) and excysting parasites are shown as Fragments per kilobase per million mapped reads (FPKM). The 789 genes to which ≥20 AS small RNAs mapped in any timepoint had very low expression under all timepoints (red), compared to expression of all genes (blue), indicating that they are not regulated during development.

We compared ORFs with ≥20 AS small RNAs from each of the developmental time-points and found significant overlap (Fig 5A). Of the 789 ORFs with ≥20 AS RNAs in at least one time-point, 705 met the same criteria for at least two other time points, while only 32 ORFs were unique to a single time-point. Further examination of the 32 unique ORFs showed that all had some small RNAs in all time-points, but simply a lower number than the 20 small RNA cutoff, suggesting that these differences are most likely an artifact of differing library sizes. Using previously generated RNA-Seq data [7], we found that median expression of the 789 ORFs was very low throughout development; thus the genes to which ≥20 small RNAs map appear to be constitutively silenced and are not transcriptionally regulated during development (Fig 5B). Based on our previously published RNA-Seq data, only 12 of these 789 ORFs has significant differential mRNA expression between any of the two time points observed.

To test whether our observation was an artifact of the relatively low cutoff of ≥20 mapped AS small RNAs, we repeated this analysis with a cutoff of ≥50 AS small RNAs; however, the results were very similar. Of the 595 genes with ≥50 AS small RNAs at any stage, virtually all were not expressed at all time points. Our previous work on the transcriptome of *Entamoeba* stage conversion identified over 4,000 genes with significantly changed expression between trophozoites, early cysts mature cysts and excysting parasites [7]. Thus, the lack of changes in 27nt small RNAs during development was unexpected, given the vast changes in gene expression and cell physiology that occur during this time. These data indicate that the 27nt small RNAs in *E*. *invadens* are likely not involved in regulation of gene expression during development. Instead this small RNA population appears to permanently silence a subset of genes. Permanent gene silencing by the 27nt small population has been noted in *E*. *histolytica* in the G3 strain, where the RNAi pathway intersects with epigenetic maintenance of gene silencing, which appear to be un-reversible [20, 37, 38].

### Sequencing of small RNA libraries from *E*. *histolytica* under basal and stress conditions

**Fig 6:**
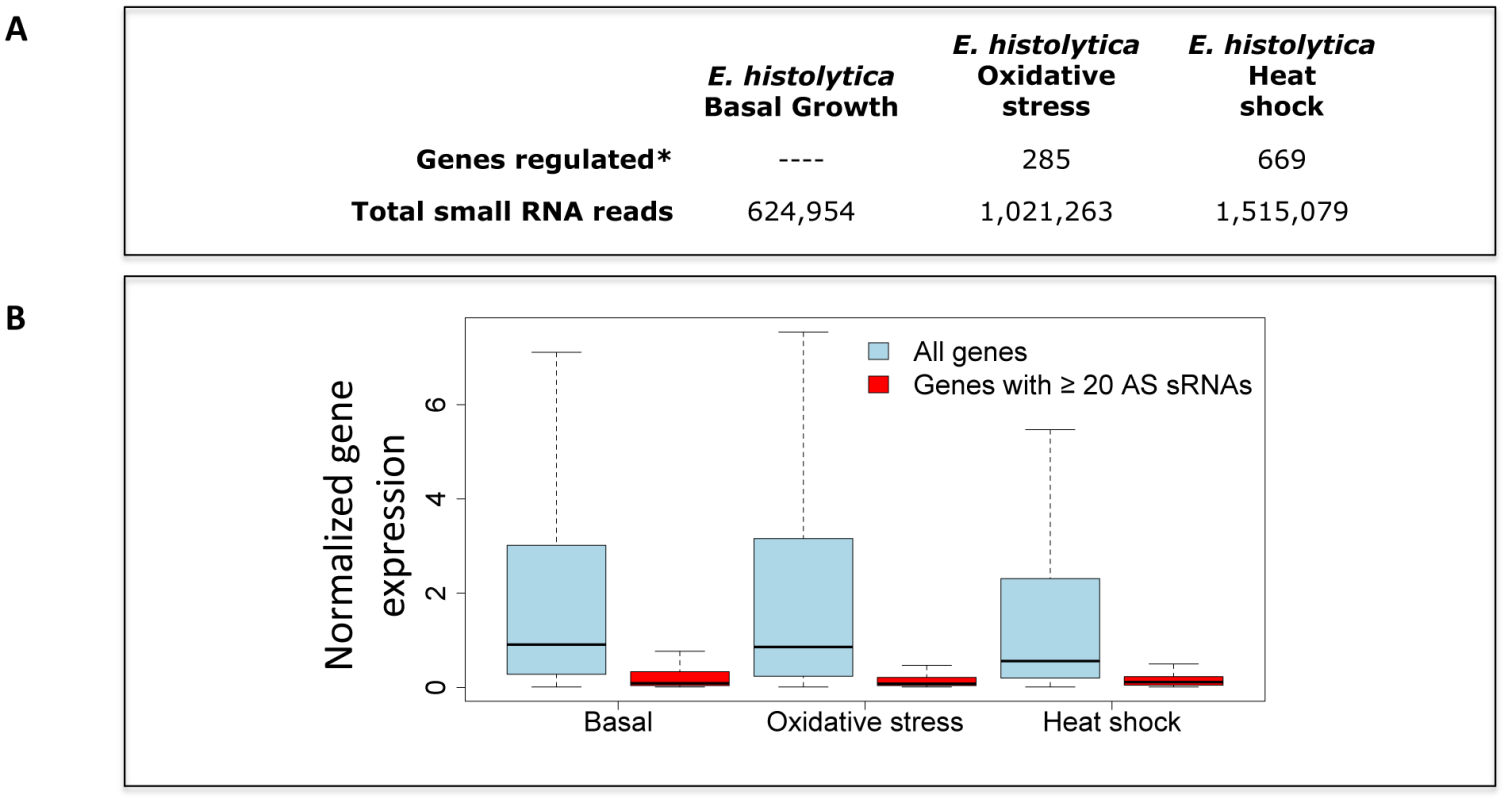
*E*. *histolytica* small RNA libraries and comparison of genes with abundant small RNAs. **(A)** Number of genes regulated and total small RNA reads analyzed for each *E*. *histolytica* condition listed. Genes regulated during oxidative stress adapted from [9] and defined as genes with an expression change that is 2-fold, p-value < 0.05. For heat shock data were adapted from [8] and defined as expression change 2-fold, p-value < 0.05. Small RNA reads are total reads prior to size selection and removal of duplicates. **(B)** Genes with ≥20 AS small RNAs are not expressed under basal or stress conditions. Normalized microarray data from *E*. *histolytica* trophozoites, trophozoites after exposure to H_2_O_2_, and trophozoites after heat shock are shown. The 316 genes to which ≥20 AS small RNAs map in any condition had very low expression under all conditions (red), indicating that they are not regulated by stress.

Since the 27nt small RNAs did not seem to be playing a role in regulating *Entamoeba* stage conversion, we wanted to determine if this population controls gene expression during stress response. We have previously cataloged transcriptional changes in *E*. *histolytica* after heat shock and under oxidative stress [8, 9]. We therefore chose those same conditions (42°C for 1hr or 1mM H_2_O_2_ for 1hr) to treat *E*. *histolytica* trophozoites, and generated 27nt small RNA libraries from these stress-treated parasites. Additionally, we prepared small RNA from trophozoites grown under basal conditions using identical library and sequencing conditions to allow a direct comparison. The small RNA libraries were sequenced and processed as above, and aligned to the *E*. *histolytica* genome including repetitive elements and annotated transcripts. Total number of reads sequenced for each condition are shown in Fig 6A.

In basal conditions, a total of 624,954 reads were sequenced, of which 242,277 were unique (~40%). Similar sized libraries were obtained from the heat shock and oxidative stress conditions (477,907 and 342,164 unique reads, respectively). Overall, the mapping results recapitulated our earlier results from smaller scale pyrosequencing with ~60% of mapped reads originating from ORFs, the majority of these in the antisense orientation (Table 3). As in *E*. *invadens* development, the percent of unique reads found to map to the tRNA (~0.6%), rDNA (~4%), transposons (~4.5%), or transcripts (~34%) did not change under either stress condition. Additionally, we checked the length distribution and nucleotide composition of the small RNAs isolated from *E*. *histolytica* under heat shock and oxidative stress, and found similar structure to the small RNAs from unstressed cells (peak at 27nt and 5'G bias) (S5 Fig).

**Table 3:** Composition of small RNA libraries from *E*. *histolytica* under basal and stress conditions. Sequenced libraries from each condition were processed to remove barcodes, size selected for reads >15nt or <40nt, duplicate reads removed and the remaining unique reads sequentially mapped against each sequence type shown. Note that a small number of reads mapped both antisense and sense to transcripts, hence the total number of reads mapped to “transcript” is lower than the total number of reads mapping antisense plus sense. Total number of reads and percent of unique mapped reads are shown.

Library	Basal growth	% of unique reads	Oxidative stress	% of unique reads	Heat shock	% of unique reads
**Total reads**	**624,954**		**1,021,263**		**1,515,079**	
**Unique reads**	242,277		342,164		477,907	
**Map to tRNA**	1,456	0.6%	2,137	0.6%	2,320	0.5%
**Map to rDNA**	10,438	4%	14,191	4%	18,553	4%
**Map to TEs and REs**	10,775	4%	16,258	5%	21,848	5%
**Map to genome**	**117,402**	**49%**	**166,493**	**49%**	**219,524**	**46%**
**Map to transcripts**	**83,942**	**35%**	**118,451**	**35%**	**154,727**	**32.4%**
**Map antisense to genes**	61,098	25%	85,034	25%	110,778	23.2%
**Map sense to genes**	23,064	10%	33,797	10%	44,550	9.3%
**Map to intergenic regions**	33,460	14%	48,042	14%	64,797	14%

To identify evidence of small RNA-mediated gene regulation, we looked at gene expression and 27nt small RNA abundance under heat shock and oxidative stress response. Similar to the situation in *E*. *invadens* stage conversion we saw minimal variation in the ORFs with ≥20 AS small RNAs in untreated, H_2_O_2_ treated and heat shocked parasites. Of the 8,333 annotated ORFs in the *E*. *histolytica* genome, 316 have ≥20 small RNAs map to them and 311 of the 316 ORFs met the criteria of all three conditions. We examined the microarray data on expression of these ORFs [8, 9] and found that all these genes have very low expression in all conditions (Fig 6B), with only a small number of genes (11 for heat shock and 7 for oxidative stress) having any changes in gene expression. Overall, 853 genes has significant expression changes during oxidative and heat shock stress; 17 of these had small RNAs mapping to them. We therefore conclude that *Entamoeba* 27nt small RNAs do not play a significant role in regulating gene expression during parasite stress response, despite the significant transcriptional changes that occur.

We then tested if genes targeted by AS small RNAs were conserved between *E*. *histolytica* and *E*. *invadens* genomes. Protein sequences of *E*. *invadens* genes with ≥20 AS small RNAs (789 genes) were BLAST-ed against the protein sequences of *E*. *histolytica* genes with ≥20 AS small RNAs (316 genes). However, there was no significant overlap, with only 18 *E*. *histolytica* proteins identified to be conserved (e-value < 1e^-20^), most belonging to either the β-amylase or DNA polymerase gene families. This lack of conservation between the small RNA targeted genes may imply that the RNAi pathway performs different functions in different *Entamoeba* species, or may simply be a reflection of the sequence divergence between *E*. *histolytica* and *E*. *invadens*.

### Testing genome-wide small RNA population changes in stage conversion and stress response

To further test whether differences during development or stress could be found in small RNA populations, we compared the *E*. *invadens* trophozoite and 72h cyst libraries, as well as the *E*. *histolytica* basal, oxidative stress and heat shock libraries using Cufflinks [39]. This is the same method we used to identify the transcriptional changes during stage conversion for RNA-seq study [7] and allows for identification of regions of the genome with clusters of mapped small RNAs, whether they are in coding or intergenic regions. For the trophozoite-cyst comparison, only nine loci were identified as having differential mapping of small RNAs; none of these loci were in coding regions. Similar results were seen in *E*. *histolytica*, with only seven loci showing significant differences with oxidative stress and eight loci showing differences with heat shock. These results confirm our previous analysis indicating that very few changes in small RNA population either during stage conversion or stress.

## Discussion

Small RNAs, including small interfering RNAs and micro RNAs, play a critical role in gene regulation across most eukaryotic species. Here, we present the first survey of small RNAs from the protozoan parasite *Entamoeba invadens*, which serves as a model system for studying stage conversion in the important human pathogen *E*. *histolytica*. We determined that the *E*. *invadens* 27nt small RNA population has a 5' polyphosphate structure and that small RNAs map to genes with low mRNA abundance, hinting strongly at a gene-silencing role. However, contrary to our expectation, the 27nt small RNA population is not regulated during stage conversion and does not change in abundance or mapping during encystation or excystation. Instead we noted a substantial abundance of small RNAs derived from intergenic regions and retrotransposons, which raises the intriguing possibility that the *E*. *invadens* small RNAs could be involved in controlling transposon activity or in establishing transcriptional gene silencing at promoter elements.

Regulatory roles of 27nt small RNAs have been proven in *E*. *histolytica* [20, 36]. We have demonstrated that genes with abundant AS small RNAs have low gene expression and that induction of AS small RNAs to a gene mediates robust and stable transcriptional gene silencing. Furthermore, in comparing virulent and non-virulent strains of *E*. *histolytica* it became clear that the 27nt small RNA population mediates silencing of some strain-specific genes including virulence genes and thus contributes to the differential virulence landscape of amebic strains. Given that the RNAi pathway is known to help regulate differentiation in other systems we fully expected to identify a regulatory role for the amebic 27nt small RNAs in *Entamoeba* development. The finding that the 27nt small RNA population does not appear to regulate expression of stage-specific genes was highly unexpected. When coupled with the observation that the 27nt small RNA population does not regulate gene expression changes during oxidative stress or heat shock, the biological roles for the RNAi pathway in *Entamoeba* remain unclear. The links between small RNA silencing and epigenetic mechanisms of gene silencing have been noted in *Entamoeba* [20, 37]. This made the lack of changes in small RNAs during *Entamoeba* stage conversion even more unexpected, given that epigenetic transitions have been linked to many developmental processes, from the large scale epigenetic reprogramming in mammalian germ cells [40] to changes in histone acetylation in parasites [41].

The conservation of RNAi pathway members across *Entamoeba* species, as well as the fact that the hundreds of genes targeted by small RNAs are maintained in the genome without degeneration to pseudogenes, indicate that silenced genes are likely playing a biological role under some yet undiscovered condition. One possibility is that small RNA populations are altered during tissue invasion when the parasite invades the colon or liver. Samples from amebic colitis and liver abscess have been analyzed for transcriptomics data [42, 43]. Unfortunately, the parasite material from both colitis and liver abscess is very limited and not easily amenable for small RNA analysis; however, technical advances in small RNA library construction from highly limited biological samples may allow this avenue of investigation in the future. Another potential role for the small RNA population is a role in intercellular communication as has been noted in multiple other systems. The parasitic nematode *Heligmosomoides polygyrus* has been shown to transfer miRNAs, which regulate gene expression to a mammalian host [44]. Thus, the amebic small RNA population may mediate parasite-parasite or host-parasite intercellular communication.

Despite the fact that the 27nt small RNA population does not regulate stage conversion in *E*. *invadens*, the data will be a useful resource for the *Entamoeba* community. A number of recent publications have described transcriptional, metabolic, and proteomic changes during stage conversion [6, 7, 45, 46]. These efforts have identified numerous developmentally regulated genes, including many with predicted functions (transcription factors, signaling molecules) that indicate they could play a role in regulating stage conversion. However, functional genetic characterization in *E*. *invadens* has been hampered by the lack of genetic tools. In *E*. *histolytica* we were able to leverage our observations of the small RNA pathway to develop a powerful tool for analyzing gene function based on trigger-mediated gene silencing [21, 36]. The data in this manuscript should allow for development of a similar approach in *E*. *invadens*. Tools for regulated gene expression in *E*. *invadens* will be an important advance and allow dissection of the molecular signals that control parasite developmental biology.

## Supporting Information

S1 Fig Schematic for small RNA reads processing and alignment. A flowchart of the methods used to generate final sequence alignments is shown.
(TIFF)

S2 Fig Expression of EiAGO2-2 during development. RT-PCR showing transcript levels for EiAGO2-2 in trophozoites, early (24h) cysts, mature (72h) cysts and excysting parasites (8h). Samples prepared with and without reverse transcriptase (RT) are shown. A band for AGO2-2 is present in each timepoint; however expression seems to decrease in the cyst samples. The hypothetical protein EIN_192230, which does not change expression during development was used as a loading control. Four developmentally regulated genes: EIN_040930, EIN_099680, EIN_186850 and EIN_202650 were also included; all showed the expected changes during development.
(TIFF)

S3 Fig Distribution of small RNAs on *E*. *invadens* ORFs. **(A)** Position of first nucleotide of each alignment relative to total gene length is shown for all small RNAs that map to genes with ≥20 antisense small RNAs. **(B)** Position of first nucleotide of each alignment relative to total gene length is shown for antisense small RNAs that map to genes with ≥20 antisense small RNAs and ≤20 sense small RNAs. **(C)** Position of first nucleotide of each alignment relative to total gene length is shown for sense small RNAs that map to genes with ≥20 sense small RNAs and ≤20 antisense small RNAs.
(TIFF)

S4 Fig Nucleotide distribution of small RNAs from transposable elements LINEs and DDEs. Percent of each nucleotide base found at each position for all small RNAs from the trophozoite library that aligned to *E*. *invadens* LINE and DDE elements are shown.
(TIFF)

S5 Fig Small RNA length and nucleotide distribution of *E*. *invadens* during development and *E*. *histolytica* during stress. A profile of length distribution and the percent of each nucleotide base found at each position were calculated for each library after removal of duplicates and size selection to remove RNAs >15nt or <40nt. Note the strong peak at 27nt in length and the preference for G at the 5' end in all datasets. **(A)** *E*. *invadens* early encystation and excystation libraries. **(B)** *E*. *histolytica* basal, oxidative stress and heat shock libraries.
(TIFF)

S1 Table Small RNAs derived from transposons and repetitive elements during development. Mapping of *E*. *invadens* small RNAs to transposons and repetitive elements. Unique reads from *E*. *invadens* trophozoites, early cysts (24h), mature cysts (72h) and excysting cells (8h), after removal of tRNA and rRNA mapped reads, were aligned to each type of transposable and repetitive element found in the *E*. *invadens* genome. Total read number, percent of unique reads, and percent of the *E*. *invadens* genome that each element represents (adapted from [30]) are shown.
(XLSX)
