# Supplementary figures and images for: High Throughput Sequencing of Entamoeba 27nt Small RNA Population Reveals Role in Permanent Gene Silencing But No Effect on Regulating Gene Expression Changes during Stage Conversion, Oxidative, or Heat Shock Stress
Source: PLoS One. 2015 Aug 6;10(8):e0134481. doi: 10.1371/journal.pone.0134481 (PMC4527709; doi:10.1371/journal.pone.0134481)

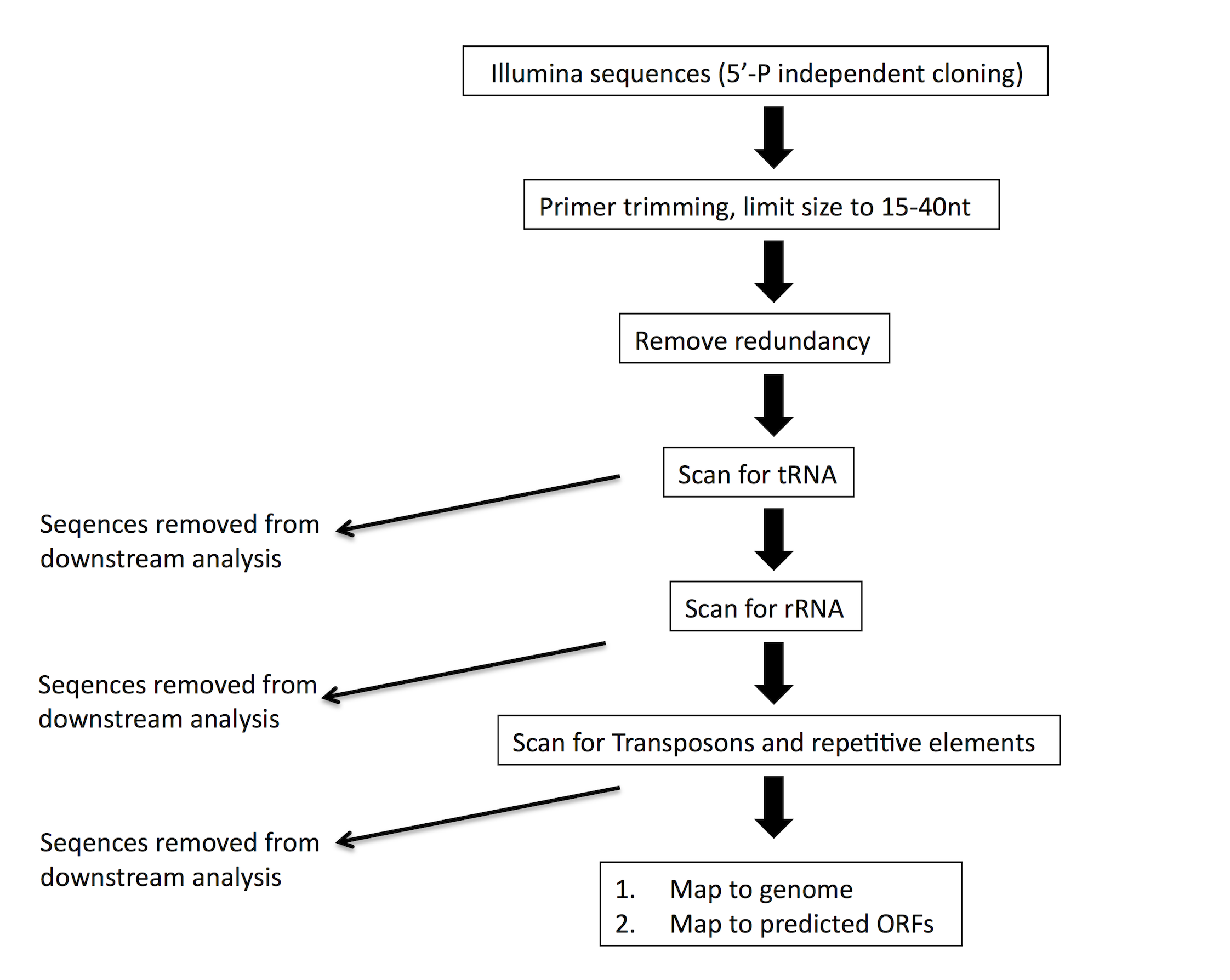

Supplement: S1 Fig — A flowchart of the methods used to generate final sequence alignments is shown. (TIFF) [file pone.0134481.s001.tiff]

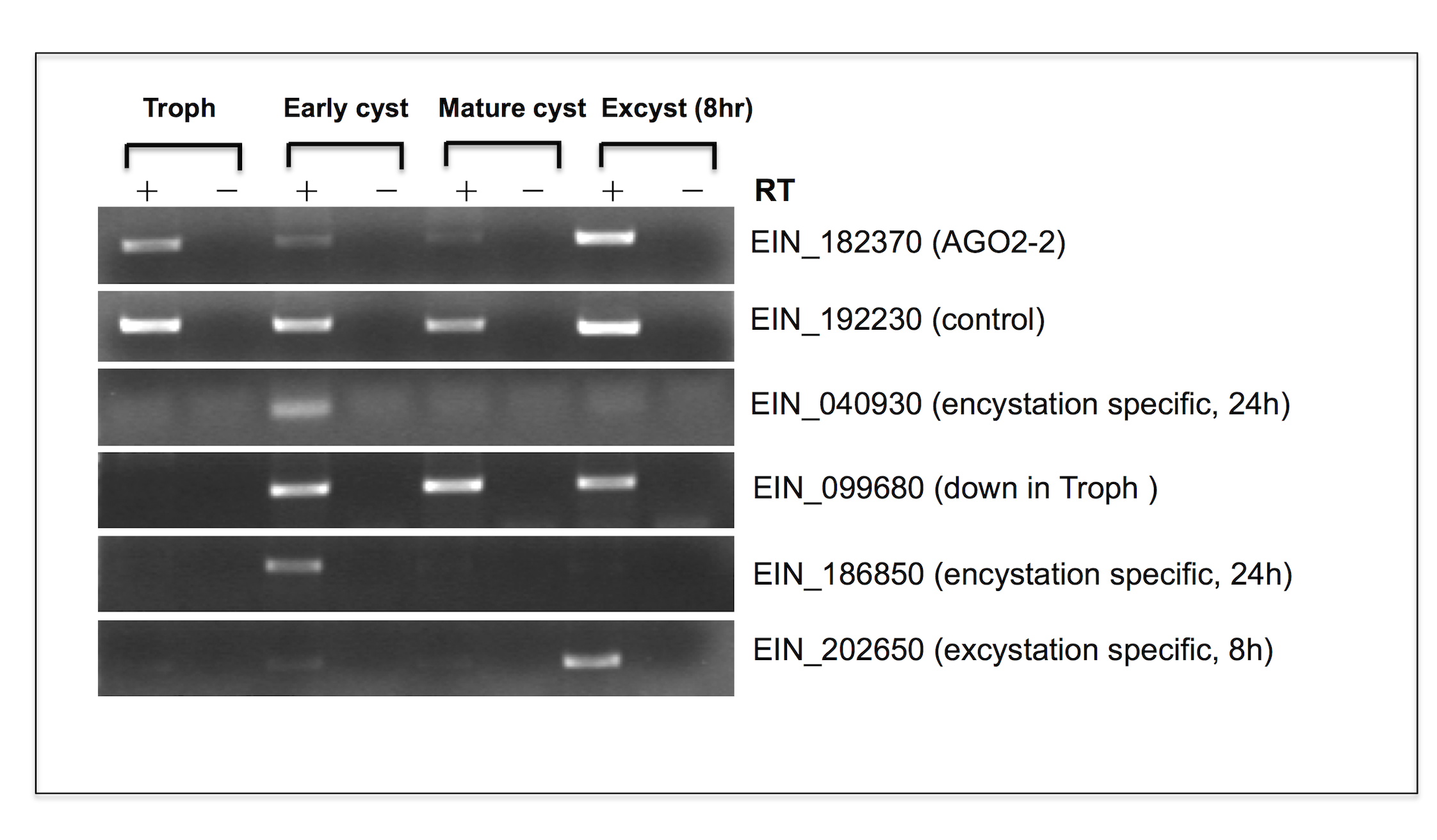

Supplement: S2 Fig — RT-PCR showing transcript levels for EiAGO2-2 in trophozoites, early (24h) cysts, mature (72h) cysts and excysting parasites (8h). Samples prepared with and without reverse transcriptase (RT) are shown. A band for AGO2-2 is present in each timepoint; however expression seems to decrease in the cyst samples. The hypothetical protein EIN_192230, which does not change expression during development was used as a loading control. Four developmentally regulated genes: EIN_040930, EIN_099680, EIN_186850 and EIN_202650 were also included; all showed the expected changes during development. (TIFF) [file pone.0134481.s002.tiff]

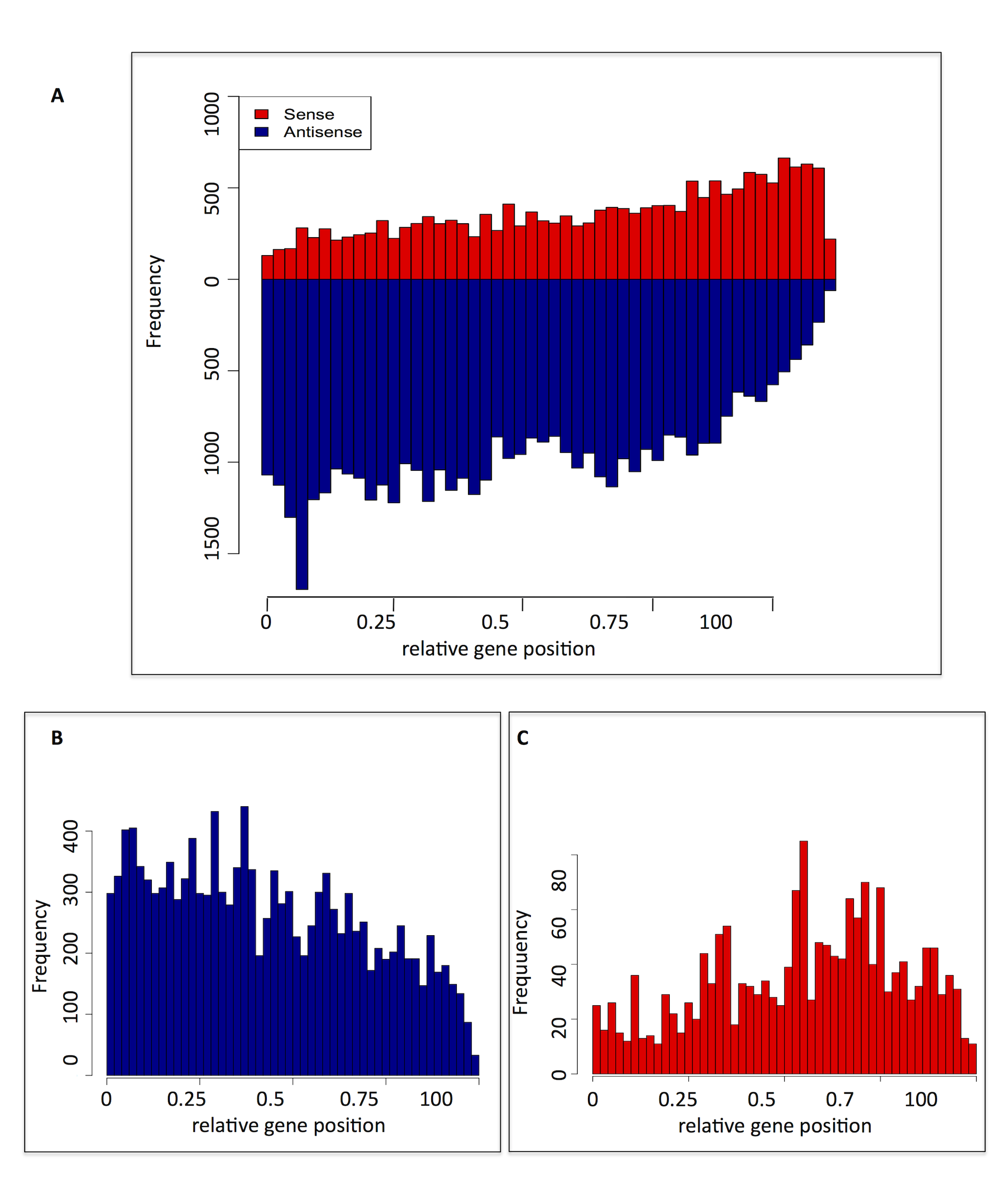

Supplement: S3 Fig — (A) Position of first nucleotide of each alignment relative to total gene length is shown for all small RNAs that map to genes with ≥20 antisense small RNAs. (B) Position of first nucleotide of each alignment relative to total gene length is shown for antisense small RNAs that map to genes with ≥20 antisense small RNAs and ≤20 sense small RNAs. (C) Position of first nucleotide of each alignment relative to total gene length is shown for sense small RNAs that map to genes with ≥20 sense small RNAs and ≤20 antisense small RNAs. (TIFF) [file pone.0134481.s003.tiff]

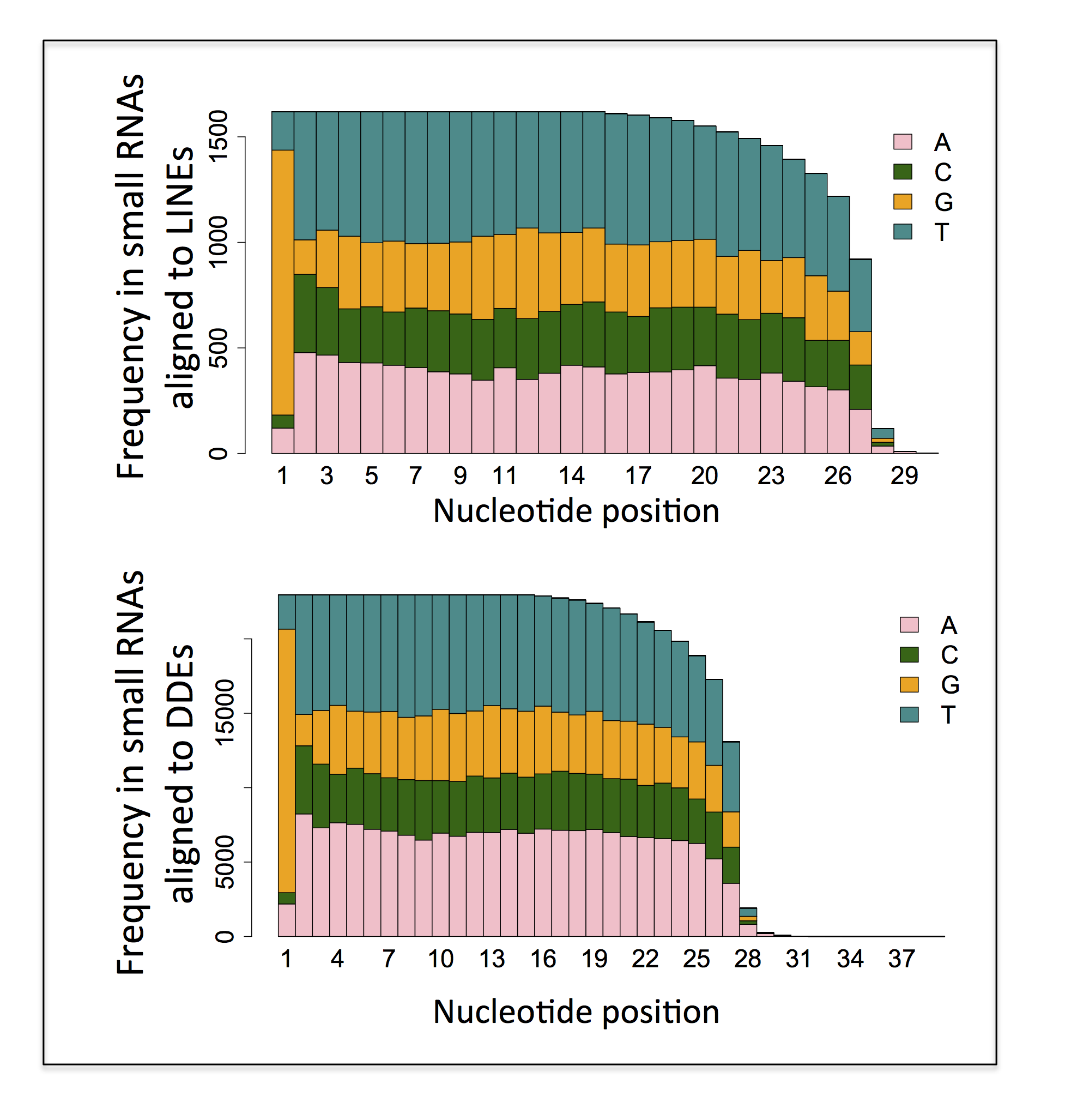

Supplement: S4 Fig — Percent of each nucleotide base found at each position for all small RNAs from the trophozoite library that aligned to E. invadens LINE and DDE elements are shown. (TIFF) [file pone.0134481.s004.tiff]

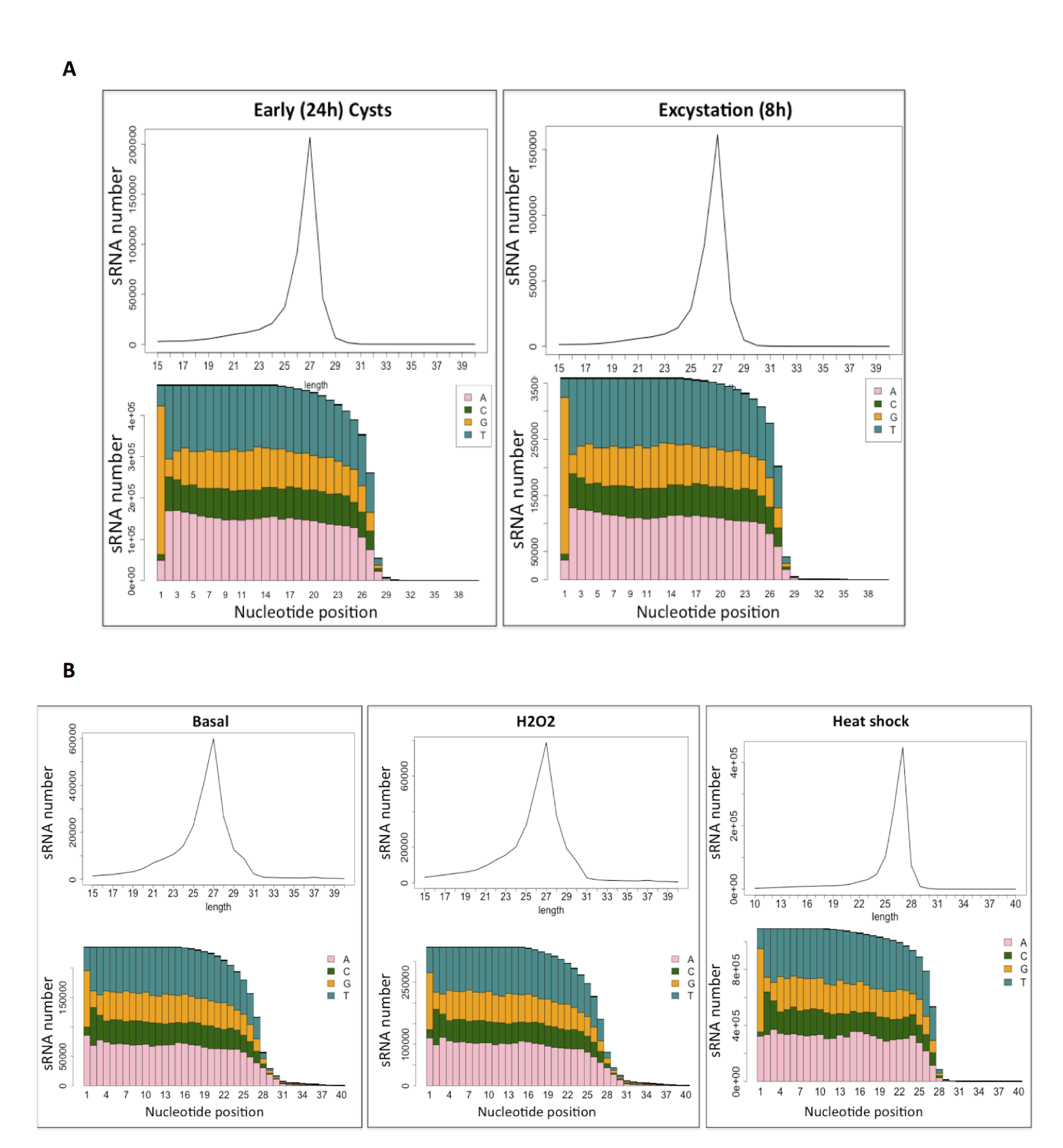

Supplement: S5 Fig — A profile of length distribution and the percent of each nucleotide base found at each position were calculated for each library after removal of duplicates and size selection to remove RNAs >15nt or <40nt. Note the strong peak at 27nt in length and the preference for G at the 5' end in all datasets. (A) E. invadens early encystation and excystation libraries. (B) E. histolytica basal, oxidative stress and heat shock libraries. (TIFF) [file pone.0134481.s005.tiff]
